# Quercetin Attenuates Pancreatic and Renal D-Galactose-Induced Aging-Related Oxidative Alterations in Rats

**DOI:** 10.3390/ijms21124348

**Published:** 2020-06-18

**Authors:** Ali H. El-Far, Mohamed A. Lebda, Ahmed E. Noreldin, Mustafa S. Atta, Yaser H. A. Elewa, Mohamed Elfeky, Shaker A. Mousa

**Affiliations:** 1 Department of Biochemistry, Faculty of Veterinary Medicine, Damanhour University, Damanhour 22511, Egypt; 2 Biochemistry Department, Faculty of Veterinary Medicine, Alexandria University, Alexandria 22758, Egypt; lebdam1979@alexu.edu.eg (M.A.L.); elfeky@alexu.edu.eg (M.E.); 3 Histology and Cytology Department, Faculty of Veterinary Medicine, Damanhour University, Damanhour 22511, Egypt; ahmed.elsayed@damanhour.edu.eg; 4 Department of Physiology, Faculty of Veterinary Medicine, Kafrelsheikh University, Kafrelsheikh 33516, Egypt; mostafa.ataa@vet.kfs.edu.eg; 5 Histology and Cytology Department, Faculty of Veterinary Medicine, Zagazig University, Zagazig 44519, Egypt; y-elewa@vetmed.hokudai.ac.jp; 6 Laboratory of Anatomy, Faculty of Veterinary Medicine, Basic Veterinary Sciences, Hokkaido University, Sapporo 060-0818, Japan; 7 Pharmaceutical Research Institute, Albany College of Pharmacy and Health Sciences, Rensselaer, NY 12144, USA

**Keywords:** aging, D-galactose, oxidative stress, quercetin, antiaging

## Abstract

Aging is an oxidative stress-associated process that progresses with age. Our aim is to delay or attenuate these oxidative alterations and to keep individuals healthy as they age using natural compounds supplementation. Therefore, we conducted the present study to investigate the protective potentials of quercetin against D-galactose (D-gal)-associated oxidative alterations that were induced experimentally in male Wistar rats. Forty-five rats were randomly allocated into five groups of nine rats each. The groups were a control group that was reared on a basal diet and injected subcutaneously with 120 mg D-gal dissolved in physiological saline solution (0.9% NaCl) per kg body weight daily and quercetin-treated groups that received the same basal diet and subcutaneous daily D-gal injections were supplemented orally with 25, 50, and 100 mg of quercetin per kg body weight for 42 days. Pancreatic and renal samples were subjected to histopathological, immunohistochemical, and relative mRNA expression assessments. Aging (*p53*, *p21*, *IL-6*, and *IL-8*), apoptotic (*Bax*, *CASP-3*, and caspase-3 protein), proliferative (Ki67 protein), antiapoptotic (*Bcl2* and Bcl2 protein), inflammatory (*NF-κB*, *IL-1β*, and *TNF-α*), antioxidant (*SOD1*), and functional markers (*GCLC* and *GCLM* genes and insulin, glucagon, and podocin proteins) were determined to evaluate the oxidative alterations induced by D-gal and the protective role of quercetin. D-gal caused oxidative alterations of the pancreas and kidneys observed via upregulations of aging, apoptotic, and inflammatory markers and downregulated the antiapoptotic, proliferative, antioxidant, and functional markers. Quercetin potentially attenuated these aging-related oxidative alterations in a dose-dependent manner. Finally, we can conclude that quercetin supplementation is considered as a promising natural protective compound that could be used to delay the aging process and to maintain human health.

## 1. Introduction

Aging is a deterioration process with reduced physical and functional potential due to oxidative stress, which leads to increased risk of stress-related oxidative diseases [1]. The aged person is vulnerable to oxidative stress-induced cell damage due to reduced antioxidant ability and the accumulation of reactive oxygen species (ROS) and oxidizing products leading to different cellular alterations [2]. A model of aging based on D-galactose (D-gal) is a widely used antiaging model, and routine and quantitative D-gal injection in rats may trigger symptoms similar to natural aging [3].

Natural compounds are commonly used for prevention of various aging changes, such as antioxidants and anti-inflammatory agents [4]. Quercetin (3,3′,4′,5,7-pentahydroxyflavone) is one of the natural compounds that has an antioxidant potential [5]. It is one of the most effective antioxidants in the flavonoid family and is found in kales, onions, berries, apples, red grapes, broccoli, cherries, and tea [6,7]. Quercetin has also been extracted from many herbs such as *Euonymus alatus*, *Nelumbo nucifera*, *Ginkgo biloba*, *Morus alba,* and *Phoenix dactylifera* [8,9,10].

Quercetin’s antioxidant function is mainly defined by its enhanced reduced glutathione (GSH) and antioxidant enzyme function via the removal of ROS [11]. Quercetin’s antioxidant capacity is attributed to the presence of the catechol group (B ring in Figure S1) and the OH group at position 3 of the AC ring (Figure S1), which have the optimal free-radical scavenging [12]. Numerous studies have stated the antioxidant potential of quercetin. Darband et al. [13] stated that quercetin mitigated oxidative harms in rats treated with 1,2-dimethylhydrazine through the nuclear factor erythroid 2-related (Nrf2) signaling pathway during colon cancer induction. Also, the serum superoxide dismutase (SOD) and GSH concentrations in streptozotocin-induced diabetic rats were significantly enhanced by quercetin and gliclazide combination [14]. In rats with full Freund’s adjuvant-induced arthritis by reduction of reactive substances in thiobarbituric acid reactive substances and ROS, quercetin secured against oxidative stress [15]. For these antioxidant potentials of quercetin, we undertook the current study to investigate its antiaging protective effects on D-gal-treated male rats.

## 2. Results

### 2.1. Pancreatic Histomorphology 

**Figure 1:**
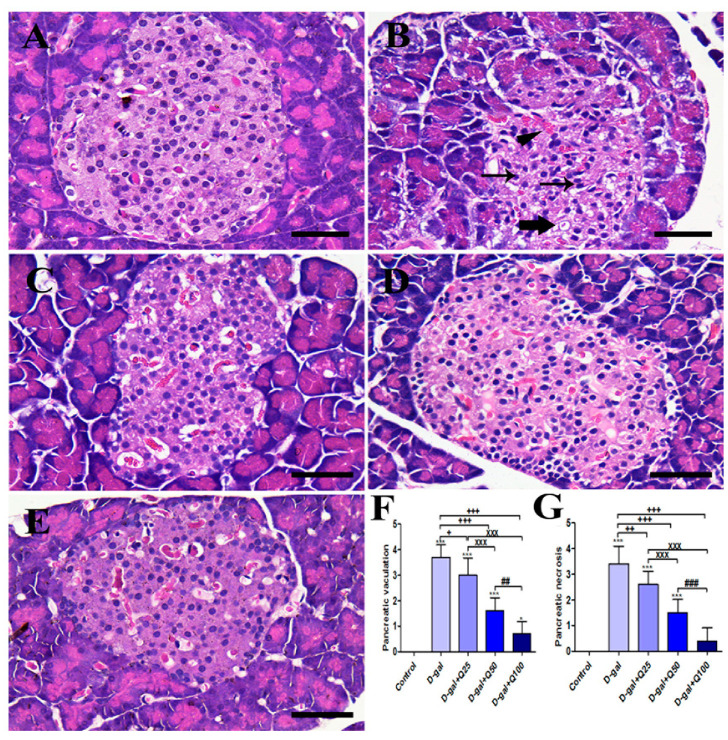
Histopathological examination of rats’ pancreas. (**A**) Negative control group. (**B**) D-gal group revealing pyknotic nuclei (thin arrows), congested blood vessels (arrowhead) and vacuolations in cells (thick arrow). (**C**) D-gal+Q25 group. (**D**) D-gal+Q50 group. (**E**) D-gal+Q100 group. (**F**) Hematoxylin and Eosin (H&E) semiquantitative scoring of pancreatic vacuolations. (**G**) H&E semiquantitative scoring of pancreatic necrosis. Scale bar = 50 µm. Data were analyzed with one-way ANOVA followed by Tukey’s multiple comparison test. * *p* < 0.05 and *** *p* < 0.001 vs. control. ^+^ *p* < 0.05, ^++^ *p* < 0.01, and ^+++^ *p* < 0.001 vs. D-gal. ^xxx^ *p* < 0.001 vs. D-gal+Q25. ^##^ *p* < 0.01 and ^###^ *p* < 0.001 vs. D-gal+Q50. Error bars represent mean ± SD.

The negative control group showed the typical pancreatic architecture that consisted of uniform pancreatic exocrine acini with normal islets of Langerhans (Figure 1A). On the other hand, the islets of Langerhans are shrunken and have many vacuolated cells and pyknotic nuclei in the D-gal group (Figure 1B). The D-gal+Q25 group showed improvement of the islets of Langerhans outlines with lower vacuolated cells and lower necrosis than the D-gal group (Figure 1C). The D-gal+Q50 group revealed a relatively healthy pancreatic structure like the negative control group (Figure 1D). The D-gal+Q100 group showed the best protection of the pancreatic architecture (Figure 1E). Semiquantitative statistical analysis of the pancreatic lesion scores showed that the animals treated with D-gal had significantly higher pancreatic vacuolations and necrosis scores than rats in the control group. However, compared with rats in the D-gal group, the D-gal+Q25, D-gal+Q50, and D-gal+Q100 groups showed significant reductions in the pancreatic lesions score (Figure 1F,G).

### 2.2. Pancreatic Immunohistochemistry 

**Figure 2:**
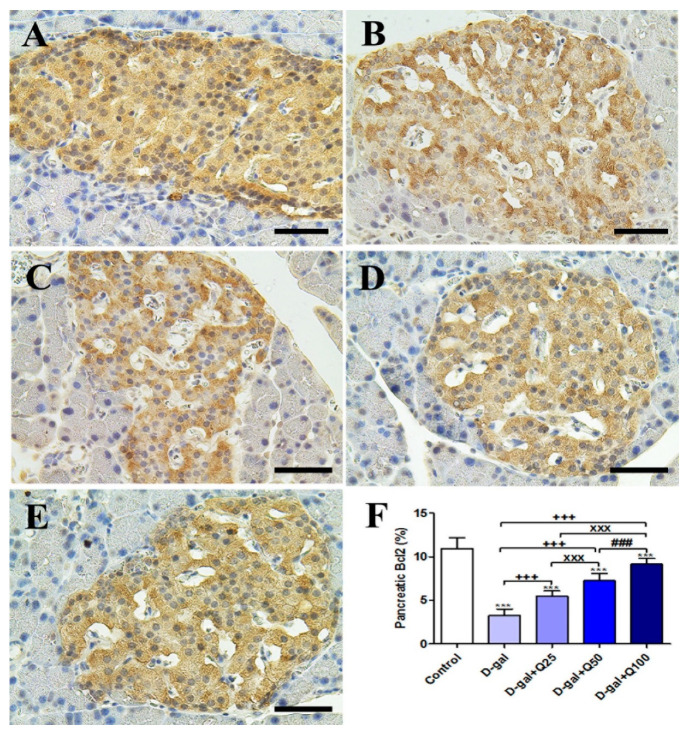
Immunohistochemical staining of rats’ pancreas with B-cell lymphoma 2 (Bcl2). (**A**) Negative control group. (**B**) D-gal group. (**C**) D-gal+Q25 group. (**D**) D-gal+Q50 group. (**E**) D-gal+Q100 group. (**F**) Quantification of Bcl2 in the pancreatic islets of Langerhans in different groups. Scale bar = 50 µm. Data were analyzed with one-way ANOVA followed by Tukey’s multiple comparison test. *** *p* < 0.001 vs. control. ^+++^ *p* < 0.001 vs. D-gal. ^xxx^ *p* < 0.001 vs. D-gal+Q25. ^###^ *p* < 0.001 vs. D-gal+Q50. Error bars represent mean ± SD.

Immunohistochemical staining of rats’ pancreas with B-cell lymphoma 2 (Bcl2) showed strong Bcl2 reaction in the islets of Langerhans in the negative control (Figure 2A). On the other hand, the D-gal group revealed a weak Bcl2 reaction in most cells of the islets of Langerhans (Figure 2B). The D-gal+Q25 showed a stronger Bcl2 reaction in most cells of the islets of Langerhans than the D-gal group (Figure 2C). The D-gal+Q50 revealed a moderate reaction in the islets of Langerhans (Figure 2D). The D-gal+Q100 group showed the strongest Bcl2 reaction in the islets of Langerhans (Figure 2E). The D-gal+Q100 group showed the strongest Bcl2 reaction in the renal tubules and renal glomeruli (Figure 2E). Semiquantitative statistical analysis of Bcl2 distribution in the pancreatic islets of Langerhans revealed a significant reduction in Bcl2 in the D-gal group. This low expression of Bcl2 due to D-gal was significantly elevated in the D-gal+Q25-, D-gal+Q50-, and D-gal+Q100-treated rats (Figure 2F).

**Figure 3:**
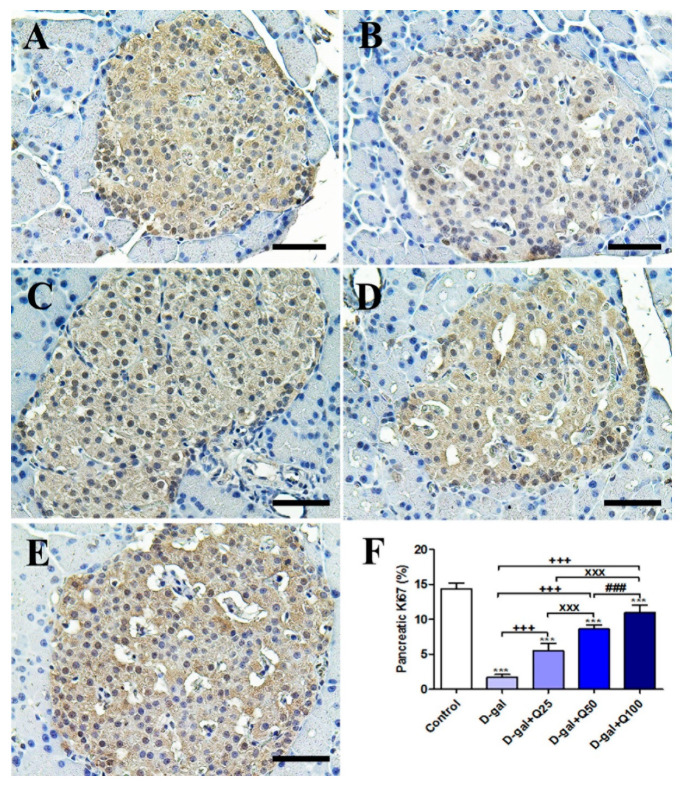
Immunohistochemical staining of rats’ pancreas with Ki67. (**A**) Negative control group. (**B**) D-gal group. (**C**) D-gal+Q25 group. (**D**) D-gal+Q50 group. (**E**) D-gal+Q100 group. (**F**) Quantification of Ki67 in pancreatic islets of Langerhans in different groups. Scale bar = 50 µm. Data were analyzed with one-way ANOVA followed by Tukey’s multiple comparison test. *** *p* < 0.001 vs. control. ^+++^ *p* < 0.001 vs. D-gal. ^xxx^ *p* < 0.001 vs. D-gal+Q25. ^###^ *p* < 0.001 vs. D-gal+Q50. Error bars represent mean ± SD.

The negative control showed the strongest Ki67 reaction in the islets of Langerhans (Figure 3A). However, the D-gal group revealed the weakest Ki67 reaction in the islets of Langerhans (Figure 3B). The D-gal+Q25 group showed stronger Ki67 reaction in the islets of Langerhans than the D-gal group (Figure 3C). The D-gal+Q25 group exhibited a moderate Ki67 reaction in the islets of Langerhans (Figure 3D). The D-gal+Q100 group showed the strongest Ki67 reaction in the islets of Langerhans (Figure 3E). Semiquantitative statistical analysis of Ki67 distribution in the islets of Langerhans revealed a significant reduction in the Ki67 expression in the D-gal group compared with control rats. This low expression in Ki67 due to D-gal was significantly elevated in the D-gal+Q25-, D-gal+Q50-, and D-gal+Q100-treated rats (Figure 3F).

**Figure 4:**
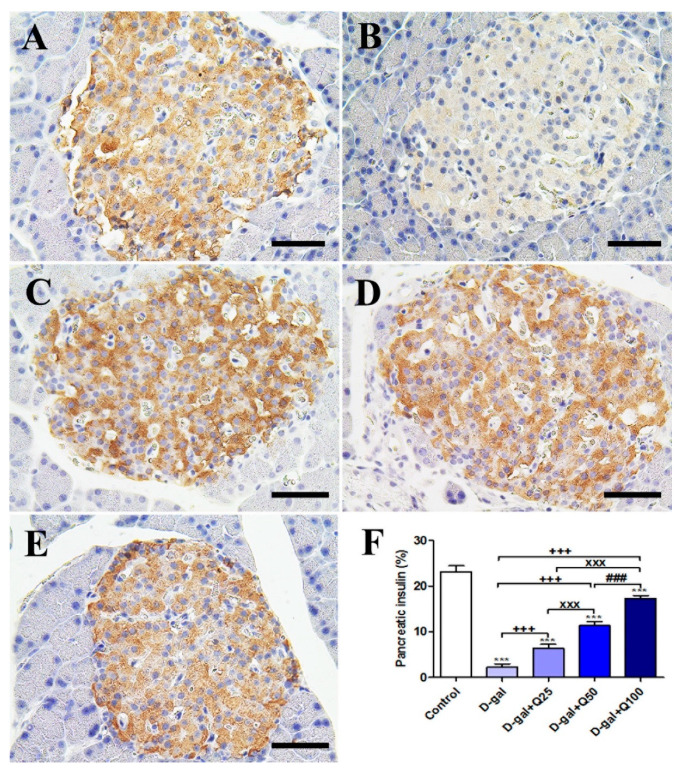
Immunohistochemical staining of rats’ pancreas with insulin. (**A**) Negative control group. (**B**) D-gal group. (**C**) D-gal+Q25 group. (**D**) D-gal+Q50 group. (**E**) D-gal+Q100 group. (**F**) Quantification of insulin in pancreatic islets of Langerhans in different groups. Scale bar = 50 µm. Data were analyzed with one-way ANOVA followed by Tukey’s multiple comparison test. *** *p* < 0.001 vs. control. ^+++^ *p* < 0.001 vs. D-gal. ^xxx^ *p* < 0.001 vs. D-gal+Q25. ^###^ *p* < 0.001 vs. D-gal+Q50. Error bars represent mean ± SD.

Immunohistochemical staining of rats’ pancreas with insulin revealed a strong insulin reaction in the islets of Langerhans of the negative control (Figure 4A). On the other hand, the D-gal group showed a weak insulin reaction in the islets of Langerhans (Figure 4B). The D-gal+Q25 group revealed a higher insulin reaction in the islets of Langerhans than the D-gal only group (Figure 4C). The D-gal+Q50 group showed a moderate insulin reaction in the islets of Langerhans (Figure 4D). The D-gal+Q100 group revealed the strongest insulin reaction in the islets of Langerhans (Figure 4E). Semiquantitative statistical analysis of insulin distribution in the islets of Langerhans revealed a significantly reduced expression of insulin in the D-gal group compared with control rats. This low expression insulin due to D-gal was significantly elevated in the D-gal+Q25-, D-gal+Q50-, and D-gal+Q100-treated rats (Figure 4F).

**Figure 5:**
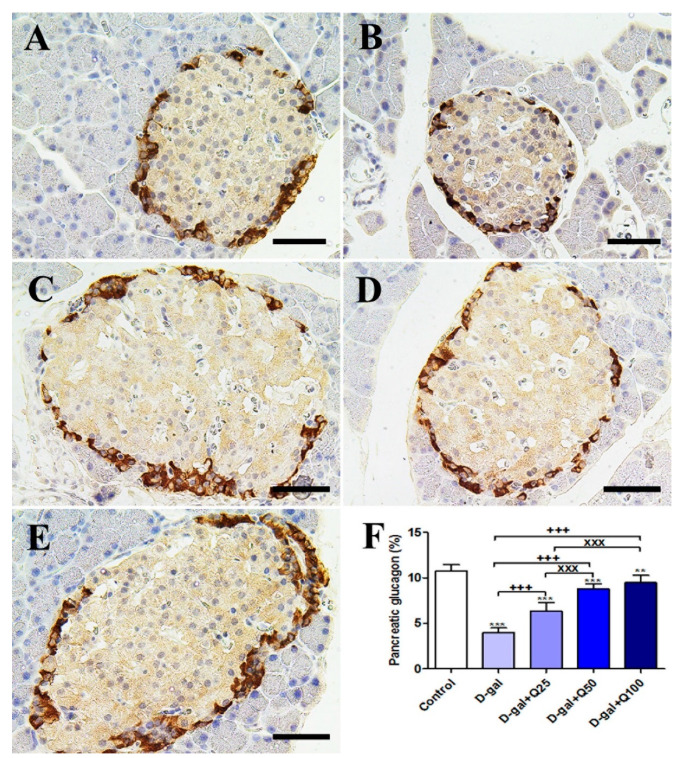
Immunohistochemical staining of rats’ pancreas with glucagon. (**A**) Negative control group. (**B**) D-gal group. (**C**) D-gal+Q25 group. (**D**) D-gal+Q50 group. (**E**) D-gal+Q100 group. (**F**) Quantification of glucagon in pancreatic islets of Langerhans in different groups. Scale bar = 50 µm. Data were analyzed with one-way ANOVA followed by Tukey’s multiple comparison test. ** *p* < 0.01 and *** *p* < 0.001 vs. control. ^+++^ *p* < 0.001 vs. D-gal. ^xxx^ *p* < 0.001 vs. D-gal+Q25. Error bars represent mean ± SD.

The negative control showed the strongest glucagon reactions in the islets of Langerhans (Figure 5A). However, the D-gal group revealed the weakest glucagon reactions in the islets of Langerhans (Figure 5B). The D-gal+Q25 group showed stronger glucagon reactions in the islets of Langerhans than the D-gal group (Figure 5C). The D-gal+Q50 group revealed moderate glucagon reactions in the islets of Langerhans (Figure 5D). The D-gal+Q100 group showed the strongest glucagon reactions in the islets of Langerhans (Figure 5E). Semiquantitative statistical analysis of glucagon distribution in the islets of Langerhans revealed a significantly reduced expression of glucagon in the D-gal group compared with control rats. This low expression in glucagon due to D-gal was significantly elevated in the D-gal+Q25-, D-gal+Q50-, and D-gal+Q100-treated rats (Figure 5F).

### 2.3. Reverse Transcription-Polymerase Chain Reaction (RT-PCR) of Pancreatic Aging, Apoptotic, Anti-Inflammatory and Antioxidant Markers 

**Figure 6:**
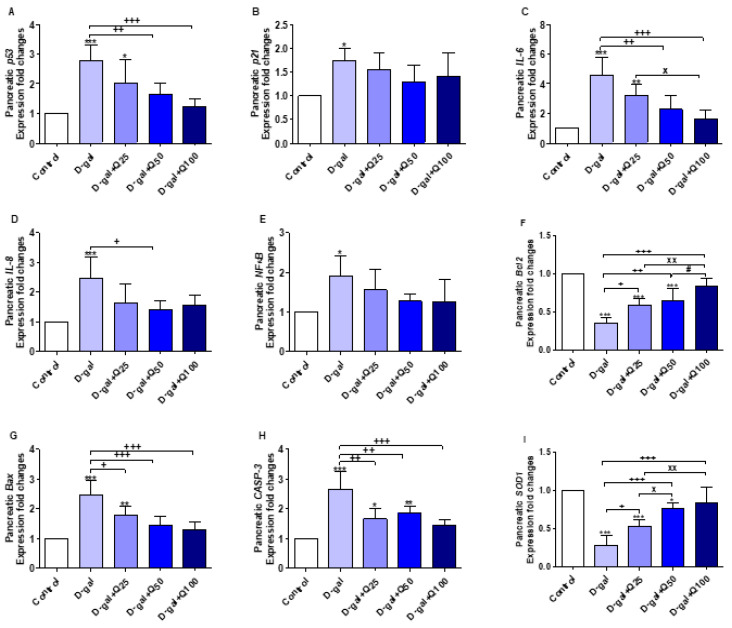
Expression of fold changes of pancreatic (**A**) *p53*, (**B**) *p21*, (**C**) interleukin-6 (*IL-6*), (**D**) interleukin-8 (*IL-8*), (**E**) nuclear factor-kappa B (*NF-κB*), (**F**) B-cell lymphoma 2 (*Bcl2*), (**G**) Bcl-2-associated X protein (*Bax*), (**H**) caspase-3 (*CASP-3*), and (**I**) Cu, Zn-superoxide dismutase (*SOD1*) mRNA levels: Data were analyzed with one-way ANOVA followed by Tukey’s multiple comparison test. * *p* < 0.05, ** *p* < 0.01, and *** *p* < 0.001 vs. control. ^+^ *p* < 0.05, ^++^ *p* < 0.01, and ^+++^ *p* < 0.001 vs. D-gal. ^x^ *p* < 0.05 and ^xx^ *p* < 0.01 vs. D-gal+Q25. ^#^ *p* < 0.05 vs. D-gal+Q50. Error bars represent mean ± SD. n = 5.

The results illustrated in Figure 6A–I represent the protective effect of quercetin against aging alterations of pancreatic tissues in response to D-gal. *p53* (*p* < 0.001) and *p21* (*p* < 0.05), the aging-related markers, were significantly increased in pancreatic tissues in the D-gal group. *p53* in aged rats treated with quercetin in D-gal+Q50 (*p* < 0.01) and D-gal+Q100 (*p* < 0.001) were significantly decreased when compared with untreated aged rats (Figure 6A).

The expression of interleukin-6 (*IL-6*) (Figure 6C) and interleukin-8 (*IL-8*) (Figure 6D) were significantly (*p* < 0.001) increased in the D-gal group. The D-gal+Q50 (*p* < 0.01) and D-gal+Q100 (*p* < 0.001) groups significantly decreased the elevated *IL-6* mRNA expression in comparison with the D-gal group. Also, D-gal+Q50-supplemented rats exhibited a significant (*p* < 0.05) decrease in *IL-8* mRNA expression compared with the D-gal.

Pancreatic nuclear factor-kappa B (*NF-κB*) expression was significantly increased in the D-gal group (*p* < 0.05) (Figure 6E), and in the same group, the anti-apoptosis-related gene, *Bcl2*, expression was significantly (*p* < 0.001) decreased (Figure 6F). The apoptosis-related genes, Bcl-2-associated X protein (*Bax*) and caspase-3 (*CASP-3*), had expressions that were significantly (*p* < 0.001) increased (Figure 6G,H). Quercetin supplementation reversed the effects of D-gal on the abovementioned anti-apoptosis and apoptosis-related genes (Figure 6E–H).

The expression fold changes of the pancreatic *SOD1* were significantly decreased in D-gal (*p* < 0.001), D-gal+Q25 (*p* < 0.001), and D-gal+Q50 (*p* < 0.05) compared with control untreated (Figure 6I). In comparison with D-gal, quercetin supplementation in D-gal+Q25 (*p* < 0.05), D-gal+Q50 (*p* < 0.001), and D-gal+Q100 (*p* < 0.001) significantly increased *SOD1* mRNA expression.

### 2.4. Renal Histomorphology 

**Figure 7:**
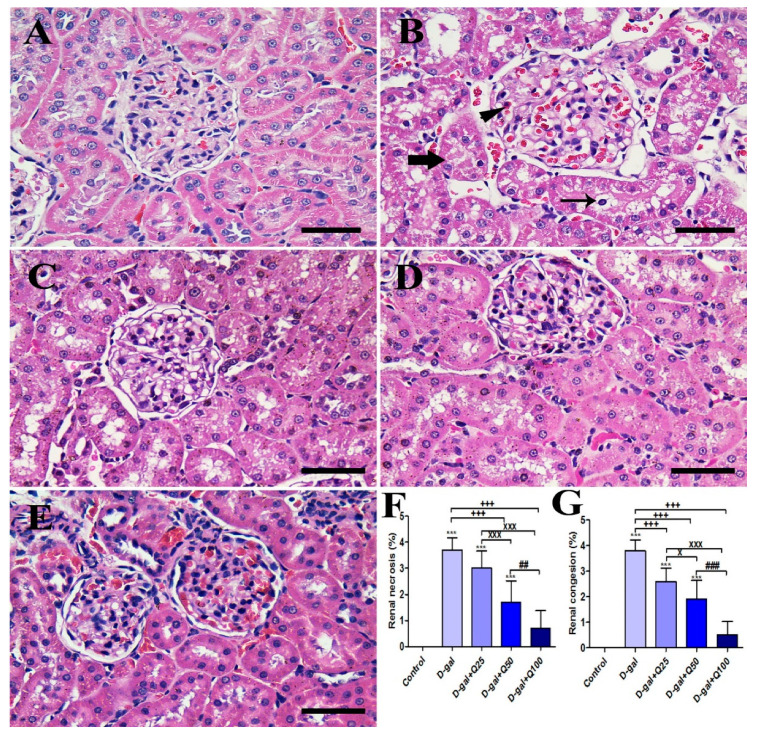
Histopathological examination of rats’ kidney. (**A**) Negative control group. (**B**) D-gal group revealing congestion (arrowhead), degeneration in the renal tubules (thin arrow), and intratubular eosinophilic proteinaceous materials inside the lumen of renal tubules (thick arrow). (**C**) Gal-Q25 group. (**D**) D-gal+Q50 group. (**E**) D-gal+Q100 group. (**F**) H&E semiquantitative scoring of renal necrosis. (**G**) H&E semiquantitative scoring of renal congestion. Scale bar = 50 µm. Data were analyzed with one-way ANOVA followed by Tukey’s multiple comparison test. Error bars represent mean ± SD. *** *p* < 0.001 vs. control. ^+++^ *p* < 0.001 vs. D-gal. ^x^ *p* < 0.05 and ^xxx^ *p* < 0.001 vs. D-gal+Q25. ^##^ *p* < 0.01 and ^###^ *p* < 0.001 vs. D-gal+Q50.

The negative control group showed a normal renal architecture that consisted of the uniform renal corpuscle with normal renal glomeruli and normal renal tubules (Figure 7A). On the other hand, the D-gal group revealed congestion of glomerular and intertubular capillaries, degenerative and necrotic changes of renal tubules, and intratubular eosinophilic proteinaceous materials (Figure 7B). The D-gal+Q25 group showed improvement of the proximal and distal convoluted tubules outlines with lower numbers of pyknotic nuclei and lower congestion than the D-gal group (Figure 7C). The D-gal+Q50 group revealed a relatively normal renal structure as the negative control group with normal renal glomeruli was outlined with regular Bowman’s capsule (Figure 7D). The D-gal+Q100 group showed the best protection of the renal architecture (Figure 7E). Semiquantitative statistical analysis of renal lesions scores showed that the animals treated with D-gal had significantly higher renal congestion and necrosis scores than rats in the control group. However, compared with rats in the D-gal group, the D-gal+Q25, D-gal+Q50, and D-gal+Q100 groups showed a significant reduction in the renal lesions score (Figure 7F,G).

### 2.5. Renal Immunohistochemistry 

**Figure 8:**
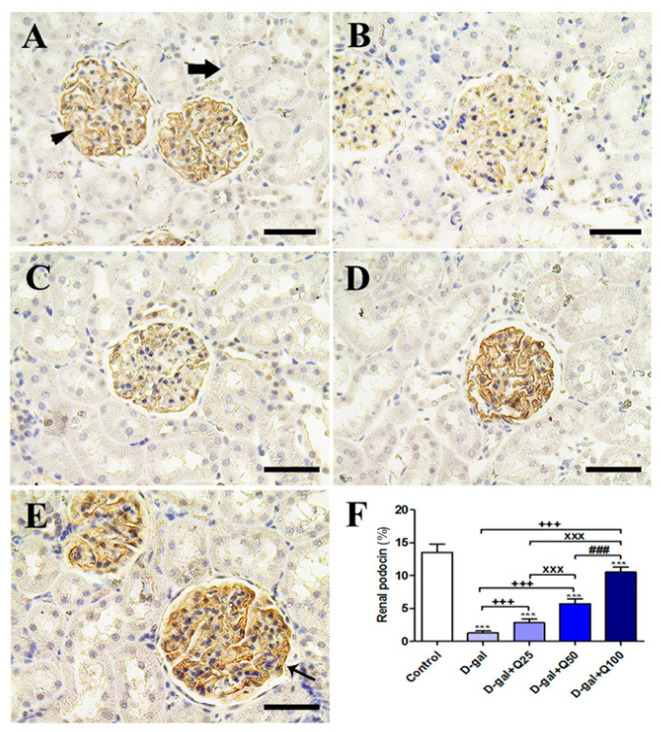
Immunohistochemical staining of rats’ kidney with podocin. (**A**) Negative control group showing high reaction in the renal glomerulus (arrowhead) and no reaction in the renal tubule (arrow). (**B**) D-gal group revealing weak podocin reaction. (**C**) D-gal+Q25 group. (**D**) D-gal+Q50 group. (**E**) D-gal+Q100 group. (**F**) Quantification of podocin in the renal tissues in different groups. Scale bar = 50 µm. Data were analyzed with one-way ANOVA followed by Tukey’s multiple comparison test. Error bars represent mean ± SD. *** *p* < 0.001 vs. control. ^+++^ *p* < 0.001 vs. D-gal. ^xxx^ *p* < 0.001 vs. D-gal+Q25. ^###^ *p* < 0.001 vs. D-gal+Q50.

Immunohistochemical staining of rat kidney with podocin (the marker of podocytes indicates the efficiency of filtration) revealed strong podocin reactions in the podocytes of the negative control (Figure 8A). On the other hand, the D-gal group showed weak podocin reactions in the renal glomeruli (Figure 8B). The D-gal+Q25 group revealed higher podocin reactions in the renal glomeruli than the D-gal only group (Figure 8C). The D-gal+Q50 group showed moderate podocin reactions in the renal glomeruli (Figure 8D). The D-gal+Q100 group revealed the strongest podocin reactions in the renal tubules (Figure 8E). Semiquantitative statistical analysis of podocin distribution in the renal glomeruli revealed a significantly lower expression of podocin in the D-gal myocytes compared with control rats. This low expression of podocin due to D-gal was significantly elevated in the D-gal+Q25-, D-gal+Q50-, and D-gal+Q100-treated rats (Figure 8F).

**Figure 9:**
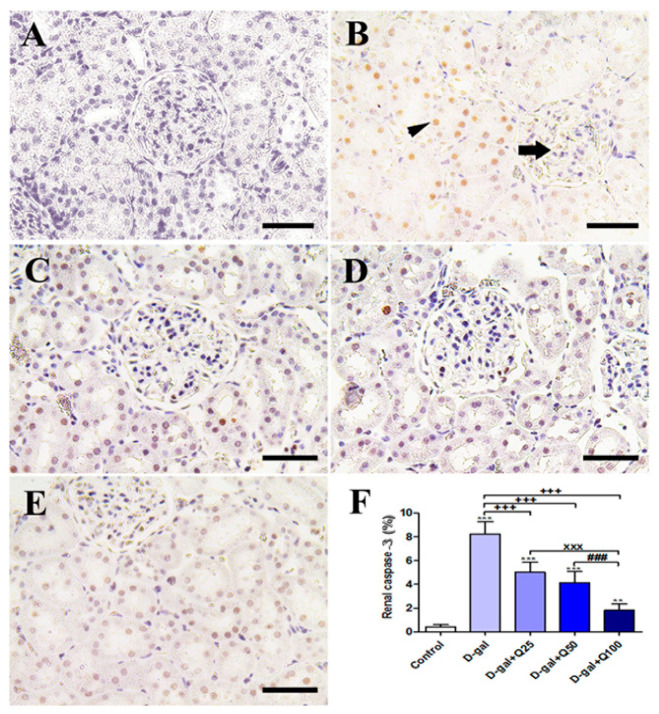
Immunohistochemical staining of rats’ kidney with caspase-3. (**A**) Negative control group. (**B**) The D-gal group revealing a strong caspase-3 reaction in the renal tubule (arrowhead) and the renal glomerulus has negative reactions (arrow). (**C**) D-gal+Q25 group. (**D**) D-gal+Q50 group. (**E**) D-gal+Q100 group. (**F**) Quantification of caspase-3 in the renal tissues in different groups. Scale bar = 50 µm. Data were analyzed with one-way ANOVA followed by Tukey’s multiple comparison test. Error bars represent mean ± SD. ** *p* < 0.01 and *** *p* < 0.001 vs. control. ^+++^ *p* < 0.001 vs. D-gal. ^xxx^ *p* < 0.001 vs. D-gal+Q25. ^###^ *p* < 0.001 vs. D-gal+Q50.

The negative control showed a negative caspase-3 reaction in all renal glomeruli and renal tubules (Figure 9A), and the D-gal group revealed the strongest caspase-3 reaction in all renal tubules with a mild reaction in the renal glomeruli (Figure 9B). The D-gal+Q25 group showed lower distribution of caspase-3-reacted nuclei in the renal tubules (Figure 9C). The D-gal+Q50 group revealed weak caspase-3 in the nuclei of renal tubules (Figure 9D). The D-gal+Q100 group showed the weakest caspase-3 reaction in the renal tubules (Figure 9E). Semiquantitative statistical analysis of caspase-3 distribution in the nuclei of the renal tubules revealed a significantly high expression of caspase-3 in the D-gal group compared with control rats. This high expression of caspase-3 due to D-gal was significantly reduced in the D-gal+Q25-, D-gal+Q50-, and D-gal+Q100-treated rats (Figure 9F).

**Figure 10:**
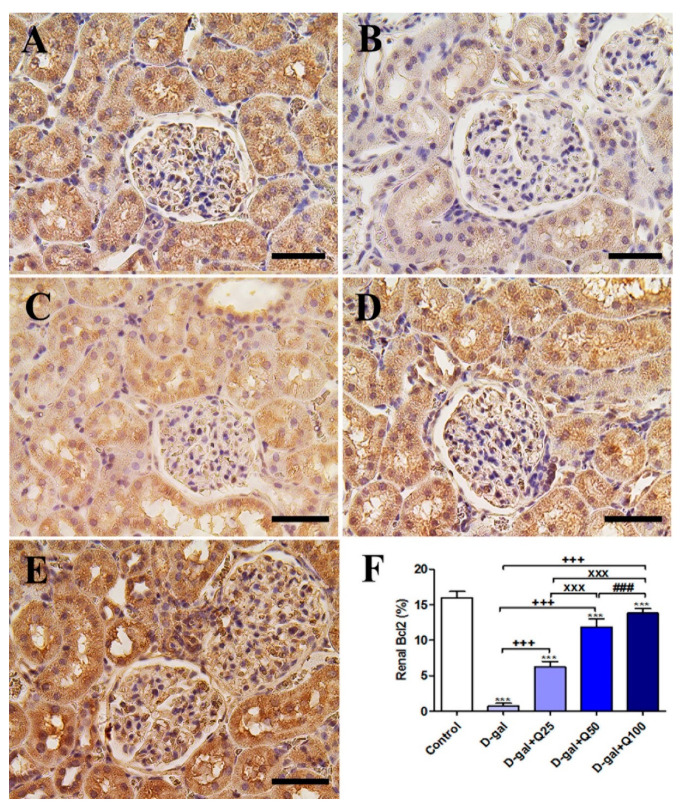
Immunohistochemical staining of rats’ kidney with B-cell lymphoma 2 (Bcl2). (**A**) Negative control group. (**B**) D-gal group. (**C**) D-gal+Q25 group. (**D**) D-gal+Q50 group. (**E**) D-gal+Q100 group. (**F**) Quantification of Bcl2 in the renal tissues in different groups. Scale bar = 50 µm. Data were analyzed with one-way ANOVA followed by Tukey’s multiple comparison test. *** *p* < 0.001 vs. control. ^+++^ *p* < 0.001 vs. D-gal. ^xxx^ *p* < 0.001 vs. D-gal+Q25. ^###^ *p* < 0.001 vs. D-gal+Q50. Error bars represent mean ± SD.

Immunohistochemical staining of rat kidney with Bcl2 showed strong Bcl2 reactions in all renal tubules and renal corpuscle in the negative control (Figure 10A). On the other hand, The D-gal group revealed weak Bcl2 reactions in most renal tubule and renal glomeruli (Figure 10B). The D-gal+Q25 group showed stronger Bcl2 reactions in the renal tubules than the D-gal group (Figure 10C). D-gal+Q50 group revealed moderate reactions in the renal tubules (Figure 10D). The D-gal+Q100 group showed the strongest Bcl2 reactions in the renal tubules and renal glomeruli (Figure 10E). Semiquantitative statistical analysis of Bcl2 distribution in the renal tubules revealed significant reduced expressions of Bcl2 in the D-gal group compared with control rats. This low expression of Bcl2 due to D-gal was significantly elevated in the D-gal+Q25-, D-gal+Q50-, and D-gal+Q100-treated rats (Figure 10F).

**Figure 11:**
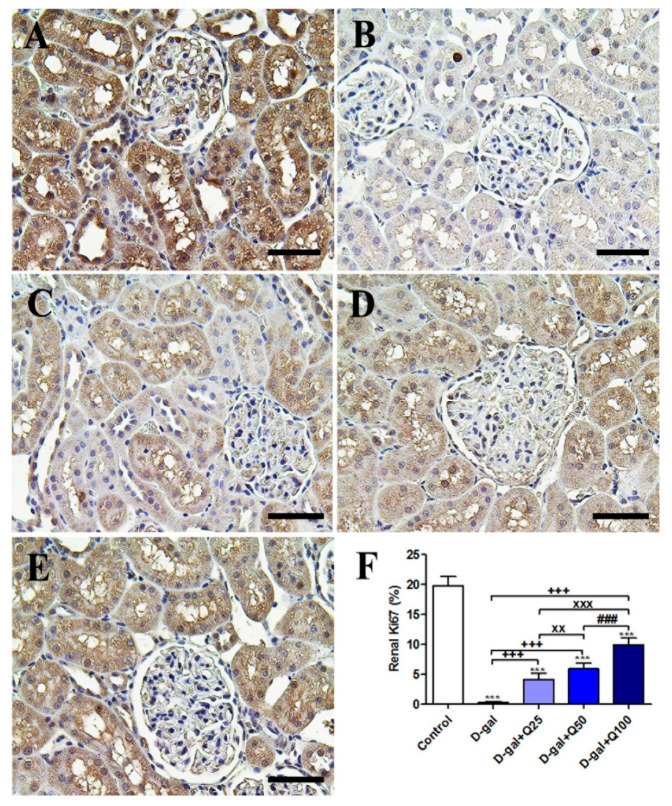
Immunohistochemical staining of rats’ kidney with Ki67. (**A**) Negative control group. (**B**) D-gal group. (**C**) D-gal+Q25 group. (**D**) D-gal+Q50 group. (**E**) D-gal+Q100 group. (**F**) Quantification of Ki67 in the renal tissues in different groups. Scale bar = 50 µm. Data were analyzed with one-way ANOVA followed by Tukey’s multiple comparison test. *** *p* < 0.001 vs. control. ^+++^ *p* < 0.001 vs. D-gal. ^xx^ *p* < 0.01 and ^xxx^ *p* < 0.001 vs. D-gal+Q25. ^###^ *p* < 0.001 vs. D-gal+Q50. Error bars represent mean ± SD.

The negative control showed the strongest Ki67 reactions in the renal tubules and renal glomeruli (Figure 11A). However, the D-gal group revealed the weakest Ki67 reactions in all renal tubules and renal glomeruli (Figure 11B). The D-gal+Q25 group showed stronger Ki67 reactions in renal tubules than the D-gal group (Figure 11C). The D-gal+Q50 group revealed moderate Ki67 reactions in the renal tubules (Figure 11D). The D-gal+Q100 group showed the strongest Ki67 reactions in the renal tubules and renal glomeruli (Figure 11E). Semiquantitative statistical analysis of the Ki67 distribution in the renal tubules revealed a significantly reduced expression of Ki67 in the D-gal group compared with control rats. This low expression of Ki67 due to D-gal was significantly elevated in the D-gal+Q25-, D-gal+Q50-, and D-gal+Q100-treated rats (Figure 11F).

### 2.6. RT-PCR of Renal Aging, Apoptotic, Anti-Inflammatory, Antioxidant and Functional Markers 

**Figure 12:**
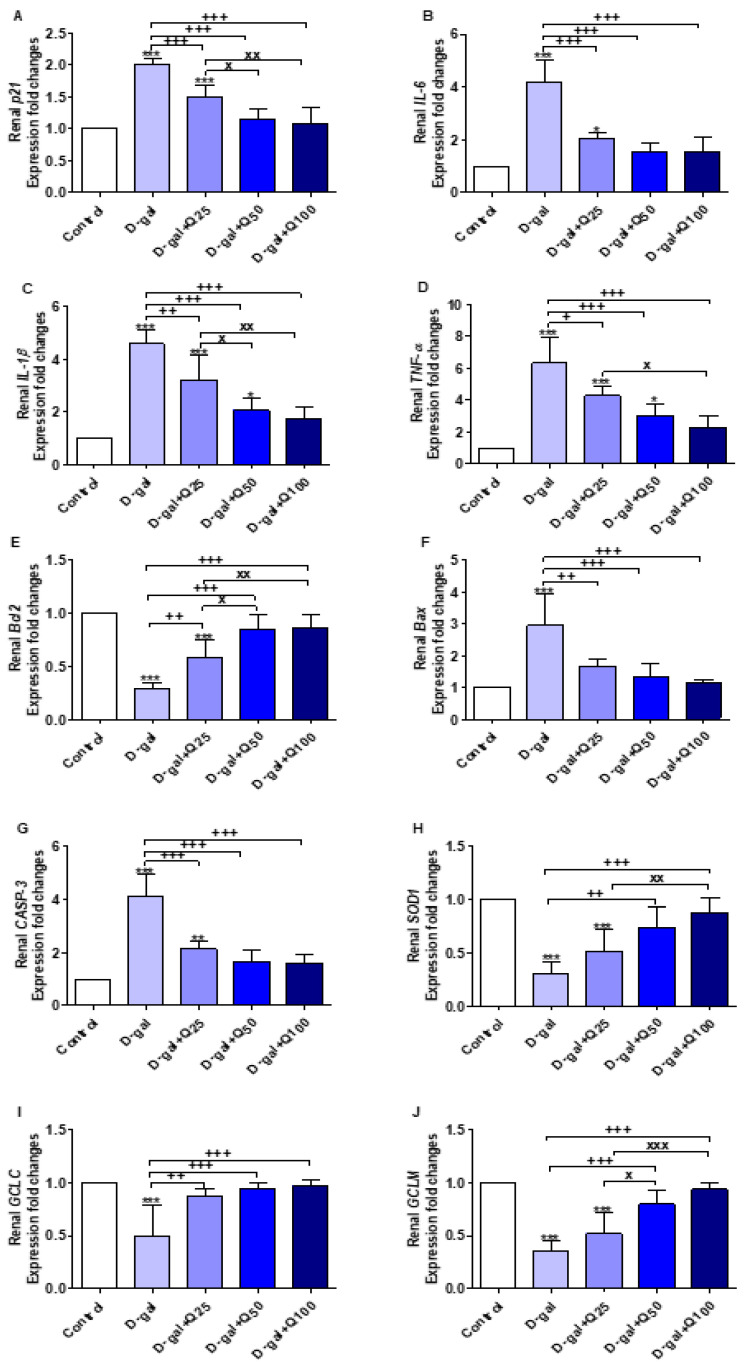
Expression of fold changes of renal (**A**) *p21*, (**B**) interleukin-6 (*IL-6*), (**C**) interleukin-1beta (*IL-1β*), (**D**) tumor necrosis factor-alpha (*TNF-α*), (**E**) B-cell lymphoma 2 (*Bcl2*), (**F**) Bcl-2-associated X protein (*Bax*), (**G**) caspase-3 (*CASP-3*), (**H**) Cu, Zn-superoxide dismutase (*SOD1*), (**I**) glutamate-cysteine ligase catalytic subunit (*GCLC*), and (**J**) glutamate-cysteine ligase regulatory subunit (*GCLM*) mRNA levels: Data were analyzed with one-way ANOVA followed by Tukey’s multiple comparison test. * *p* < 0.05, ** *p* < 0.01, and *** *p* < 0.001 vs. control. ^+^ *p* < 0.05, ^++^ *p* < 0.01, and ^+++^ *p* < 0.001 vs. D-gal. ^x^ *p* < 0.05, ^xx^ *p* < 0.01, and ^xxx^ *p* < 0.001 vs. D-gal+Q25. Error bars represent mean ± SD. n = 5.

The results illustrated in Figure 12A–J revealed significant increases in the expression of aging (*p21* and *IL-6*), inflammatory (interleukin-1beta (*IL-1β*) and tumor necrosis factor-alpha (*TNF-α*)), and apoptotic genes (*Bax* and *CASP-3*) in the D-gal group, and antiapoptotic (*Bcl2*), antioxidant (*SOD1)*, glutamate-cysteine ligase catalytic subunit (*GCLC*), and glutamate-cysteine ligase regulatory subunit (*GCLM*) were significantly decreased compared with the control. Quercetin supplementation significantly reversed the effect of the D-gal on the abovementioned genes in a dose-dependent manner.

## 3. Discussion

**Figure 13:**
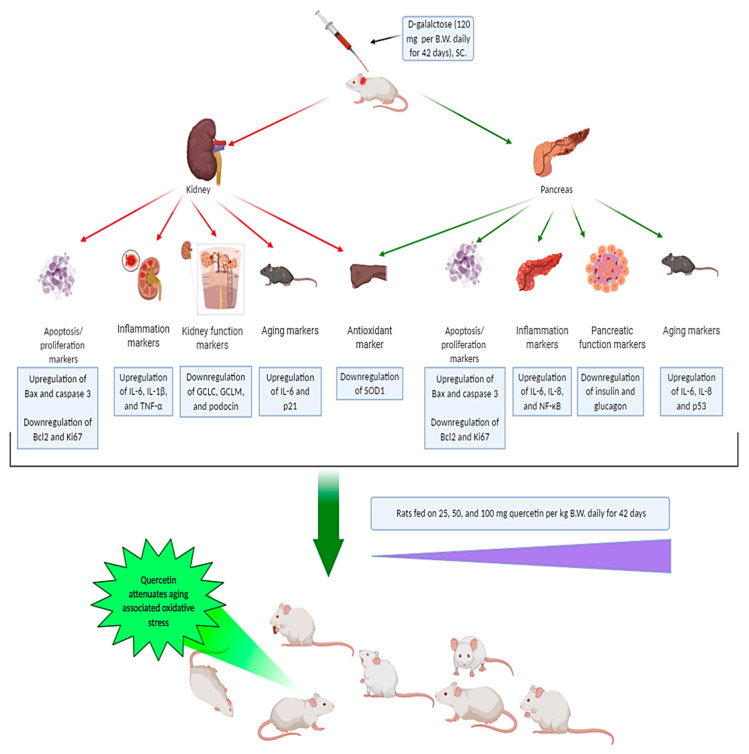
Summary of the protective effects of quercetin against oxidative stress alterations associated with aging.

ROS produced by several endogenous and exogenous processes are neutralized by cellular antioxidant defenses. Imbalance between ROS and the antioxidant defenses results in cellular oxidative stress. This imbalance occurs in the case of aging and leads to progressive loss of tissue and organ function [16]. In the current study, D-gal was injected into rats to induce aging experimentally based on oxidative stress enhancement. At the same time, quercetin was orally supplemented to investigate the antiaging protective role of quercetin against the oxidative stress associated with aging. D-gal induced oxidative alterations in the pancreatic histomorphology evidenced by significant increases in aging (*p53*, *p21*, *IL-6*, and *IL-8*), apoptotic (*Bax* and *CASP3*), and inflammatory (*NF-κB*) markers. On the contrary, the antiapoptotic (*Bcl2* and Bcl2 protein), antioxidant (*SOD1*), proliferation (Ki67), and functional (insulin and glucagon proteins) markers were significantly decreased (Figure 13). During the progression of aging, *p53* was expressed and led to expression of *p21*, which consequently induced arrest of the cell cycle and hindered cellular proliferation [17] that was shown via a significant decrease in Ki67 with production of the senescence-associated secretory phenotype (SASP) including IL-6 and IL-8. Overexpression of SASP is clear evidence for aging in addition to p53 and p21 upregulation [18]. In aged human skin fibroblasts, Wolf et al. [19] observed higher levels of IL-6 and IL-8 in the supernatant. Moreover, in aged mesenchymal stem cells and aged mice, p53 and *p21* were dramatically increased [20].

Another inflammatory marker, *NF-κB*, was significantly upregulated in response to D-gal oxidative stress. *NF-κB* has been reported to be upregulated by D-gal in numerous studies of experimentally induced aging [21,22]. However, in the present study, we demonstrated for the first time that *NF-κB* mRNA expression was upregulated in pancreatic cells in response to D-gal injection (Figure 13). Yu et al. [23] determined significant increases in brain *IL-6* and *NF-κB* in D-gal-treated mice. Hypodermic injection of D-gal in mice significantly increased the relative optical density of positive cells that were stained with senescence-associated β-galactosidase (SA-β-gal) as an aging marker in pancreatic tissue [24]. Moreover, numerous oxidative effects have been found through significant increases in p21 expression [25] and IL-6 protein levels [26] in mice and Bcl2 protein immunostaining in rats [27]. Also, Nobakht-Haghighi et al. [28] reported significant elevations in p53 and oxidative markers in pancreas of aged rats. Also, as senescence markers, the p53, p21, and p16 proteins in aged rats are considerably enhanced [29].

Apoptosis of the pancreas due to D-gal was determined through the upregulation of *Bax* and *CASP3*. In contrast, the *Bcl2* gene and Bcl2 protein content in the pancreas were significantly decreased due to the enhancement of oxidative stress and decline of the antioxidant *SOD1* gene. Similarly, Dong et al. [24] stated a significant reduction in the pancreatic antioxidant potential in D-gal-treated mice via a significant decrease in SOD activity and total antioxidant capacity. On the other hand, malondialdehyde (MDA) contents in the pancreatic homogenate were significantly increased, indicating the oxidative alterations due to D-gal.

In the histological assessment, the islets had necrosis due to D-gal, which may relate to a decrease in islets’ insulin content. The interstitial fibrosis progression is a main factor in the distortion of theh exocrine and endocrine pancreas histoarchitecture that is related to vascular, ductal insular alterations. Further, the progressive enhancement of the pancreatic adipose tissue was frequently noted in the aging rats as vacuolated cells, which is associated with the natural degenerative event of aging [30]. Our results revealed significant decrease in insulin expression in islets of Langerhans in the D-gal group, which agrees with necrosis of islets of Langerhans as revealed in the histopathological examination. Our results are in agreement with a report by Ahangarpour et al. [31], who noticed considerable decrease in pancreatic islet insulin secretion in the D-gal group in mice. On the other hand, D-gal increased serum insulin levels and insulin resistance in the brain, which leads to dementia [32]. Interestingly, our results showed lowered glucagon expression in the expression in islets of Langerhans due to D-gal. The recognition system for these sugars may be present on the cell membrane of the A cell membrane and may be responsible for mediating the glucagon secretion’s inhibiting effects [33].

D-gal induced necrosis and vacuolation of renal tissues besides the induction of renal apoptosis and inflammation due to D-gal-associated oxidative stress. Also, the degenerative effects of D-gal were observed in the renal tissues in addition to significant decreases in *GCLC* and *GCLM*. Bonegio and Lieberthal [34] found that D-gal could induce infiltration of inflammatory cells, degenerative and necrotic changes of renal tubules, and intratubular eosinophilic proteinaceous materials in kidney tissue, which result in renal failure. Park et al. [35] have also revealed that D-gal injection induces kidney inflammation in rats.

Podocytes are extremely distinguished epithelial cells that form a critical part of the glomerular filtration barrier by the slit diaphragms. Reduced density and number of podocytes are associated with proteinuria and impairment of the renal function [36]. Podocin is a vital protein to maintain the slit diaphragm’s structural integrity [37,38]. Different studies showed that, in some proteinuric glomerular diseases, podocin signals are present [39,40,41]. Our investigation revealed low expressions of podocin in the D-gal group, which indicates its adverse effect on the filtration system of the kidney.

Strong antioxidant phytochemicals play a key role in treating and preventing diseases because antioxidant and ROS activities avoid alterations of oxidative interactions that lead to aging, cancer, and multiple serious illnesses [42]. Antioxidants are widely available in fruits, vegetables, cereal grains, microalgae, and medicinal plants [42,43]. Although antioxidants act to treat or prevent various diseases, when given in overdosage, they can cause toxic effects [44]. For this reason, we used quercetin at doses of 25, 50, and 100 mg per kg body weight (B.W.) daily, in a safe range that recognized by Dunnick and Halley [45], who found no toxic lesions at 6 and 15 months with the addition of quercetin at a dose range of 40–1900 mg/kg/day in rats’ diet.

Quercetin supplementation successfully reversed the oxidative stress induced by D-gal in the current study in a dose-dependent manner, showing significant decreases in aging, oxidative, inflammatory, and proapoptotic genes, while increasing the antioxidant and antiapoptotic genes. The current study is the first report concerning the protective effect of quercetin against D-gal oxidative stress alterations in the pancreas and kidneys tissues. We found only three published studies about the protective effect of quercetin against D-gal adverse alteration on brain [46,47], hepatic, and neuronal tissues [48]. Quercetin significantly increased the expression of *Nrf2*, heme oxygenase-1, and *SOD* mRNA [47] and SOD activity in brain tissue of D-gal-treated mice [46]. Also, quercetin supplementation reversed the oxidative stress of D-gal through significant increases in SOD, catalase, glutathione peroxidase, and GSH while MDA levels were significantly decreased in hepatic and neuronal tissues in rats [48].

## 4. Materials and Methods

### 4.1. Ethics Statement

The study was endorsed by the Faculty of Veterinary Medicine Ethics Committee, University of Damanhour, Egypt (approval code, 24102018), based on the recommendations of “NIH Guide for the Care and Use of Laboratory Animals”.

### 4.2. Experimental Design

Forty-five adult male Wistar rats (140 ± 20 g) were bought from the Center of Medical Research and Services, Alexandria University, Egypt. Rats were housed in standard laboratory conditions with a 12 h light/dark cycle. Food pellets and drinking water were accessed ad libitum for rats as stated by Atta et al. [49] and as listed in Table S1. After ten days, the rats were randomly allotted into five groups (n = 9 per group in three replicates each) including control, reared on basal diet and distilled water administered by gavage along with subcutaneous injection of physiological saline solution (0.9%); D-gal, reared on basal diet and injected subcutaneously with 120 mg D-gal dissolved in saline solution per kg body weight (B.W.) daily [50]; D-gal+Q25, reared on basal diet and injected subcutaneously with 120 mg D-gal per kg B.W. daily along with oral supplementation of quercetin (Sigma-Aldrich, MO, USA) dissolved in distilled water by a dose of 25 mg per kg B.W. daily [51]; and two other quercetin-treated groups, D-gal+Q50 and D-gal+Q100, that were orally supplemented daily with 2- and 4-fold dosages of quercetin compared with D-gal+Q25, respectively, and injected with the same dose as D-gal. The experiment was done for 42 days.

### 4.3. Sampling

On day 42, rats were anesthetized with an intravenous pentobarbital injection (30 mg per kg B.W.) in each group (n = 5 per group) to ensure the sampling had to be performed properly. Pancreatic and renal samples were taken for histopathology, immunohistochemistry, and relative gene expression analyses. Parts of the pancreas and left kidney of each rat were flushed with phosphate buffer saline (PBS, pH 7.4) and fixed in 4% paraformaldehyde dissolved in PBS for 48 h for sample fixation. Other parts from the pancreas and left kidney were kept frozen at −80 °C for relative gene expression analyses.

### 4.4. Histopathological Assessment

Using the traditional technique of paraffin incorporation, the fixed specimens were dehydrated by ascending degrees of ethanol, were cleared into three xylene shifts, and were finished by paraffin embedding at 65 °C. Sections (4 µm thick) were stained with Hematoxylin and Eosin (H&E) as previously described by Bancroft and Layton [52]. Semiquantitative scoring of pancreatic and renal lesions was calculated according to Gibson-Corley et al. [16]. Briefly, lesions in 10 fields were chosen randomly from each slide for each rat and averaged. The lesions were scored in a blinded way (score scale: 0 = normal; 1 ≤ 25%; 2 = 26–50%; 3 = 51–75%; and 4 = 76–100%).

### 4.5. Immunohistochemistry

Table S2 lists antibodies, sources, dilutions, and methods for retrieval of the antigen. The immunohistochemical reactions in the pancreatic and kidney sections were investigated according to the method described by Noreldin et al. [53] and Noreldin et al. [54]. Sections as prepared for histopathological assessment were deparaffinized by xylene, rehydrated in graded alcohols, and washed by distilled water. After washing with distilled water, deactivation of endogenous peroxidase was done by 3% H_2_O_2_ in absolute methanol for 5 min at 4 °C. After washing with PBS, the nonspecific reaction was blocked with 10% normal blocking serum for 60 min at room temperature. Then, the primary antibodies were incubated at 4 °C overnight. After washing with PBS, according to the species’ primary antibody hosted, the sections were incubated with biotin-conjugated goat anti-rabbit IgG antiserum or rabbit anti-goat IgG antiserum (Histofine kit, Nichirei Corporation, Tokyo, Japan) for 60 min. Then sections were washed in PBS and followed by incubation with streptavidin-peroxidase conjugate (Histofine kit, Nichirei Corporation) for 30 min. The streptavidin-biotin complex was visualized with 3,3′-diaminobenzidine tetrahydrochloride (DAB)–H_2_O_2_ solution, pH 7.0, for 3 min. The sections were washed with distilled water, and Mayer’s hematoxylin was used as a counterstain. Micrographs of the sections were taken with Leica EC3 digital camera (Leica, München, Germany) connected to Leica DM500 microscope (Leica).

For quantification of immunostaining intensities, Image J software (National Institutes of Health, Bethesda, MD, USA) was used as stated by Sysel et al. [55]. The inverse mean density was determined as reported by Vis et al. [56] in 10 randomly chosen fields from various sections of 9 rats in each group.

### 4.6. RNA Extraction and RT-PCR

Total RNA was extracted from the samples using the manufacturer’s easy-RED Total RNA Extraction Kits (iNtRON Biotechnology, Inc., Gyeonggi-do, South Korea). Agarose gel electrophoresis was used to evaluate RNA integrity, and a NanoDrop spectrophotometer was used to assess sample quantities and purities. The first-strand cDNA was achieved using the HiSen Script cDNA (iNtRON Biotechnology, Inc.) package. Specific primers were used to amplify selected genes with glyceraldehyde 3-phosphate dehydrogenase (GAPDH) as a stable housekeeping gene (Table S3 [57,58,59,60,61,62]). mRNA expression was achieved using a real-time Strata gene MX3005P PCR (Agilent Technologies, Santa Clara, CA, USA) and TOP real TM PreMIX SYBR Green qPCR master blend (cat. RT 500, Enzynomics, Daejeon, South Korea) following the manufacturer’s instructions. The relative gene expression concentrations were evaluated using the 2^−ΔΔct^ method as described by Pfaffl [63].

### 4.7. Statistical Analyses

Data were analyzed with One-way ANOVA and Tukey’s post hoc multiple range tests using GraphPad Prism 5 (GraphPad, San Diego, CA, USA). All declarations of significance depended on *p* < 0.05.

## 5. Conclusions

The oxidative stress hypothesis remains possibly the basis of aging-associated cellular alterations. Quercetin in a dose-dependent manner potentially attenuated the oxidative stress induced by D-gal in the pancreatic and renal tissues of rats through the upregulation of antioxidant (*SOD1*), antiapoptotic (*Bcl2*), and functional markers. Also, quercetin downregulated aging (*p53*, *p21*, *IL-6*, and *IL-8*), apoptotic (*Bax* and *CASP-3*), and inflammatory (*NF-κB*, *IL-1β*, and *TNF-α*) markers. Our results suggest that quercetin successfully alleviated aging of rats’ pancreatic and renal tissues, thus rendering quercetin to be a promising natural antiaging compound.

## Supplementary Materials

Supplementary materials can be found at https://www.mdpi.com/1422-0067/21/12/4348/s1, Figure S1: Chemical structure of quercetin, Table S1: Ingredients of basal diet, Table S2: Antibodies, sources, working dilutions, and conditions for antigen retrieval (10 mM citrate buffer at pH 6.0), Table S3: Primers’ sequences for RT-PCR.

## Abbreviations

Bax.	Bcl-2-associated X protein
Bcl2	B-cell lymphoma 2
CASP-3	Caspase-3
GAPDH	Glyceraldehyde 3-phosphate dehydrogenase
GCLC	Glutamate-cysteine ligase catalytic subunit
GCLM	Glutamate-cysteine ligase regulatory subunit
GSH	Reduced glutathione
IL-1β	Interleukin-1beta
IL-6	Interleukin-6
IL-8	Interleukin-8
MDA	Malondialdehyde
NF-κB	Nuclear factor-kappa B
Nrf2	Nuclear factor erythroid 2-related factor 2
ROS	Reactive oxygen species
SOD1	Cu, Zn-superoxide dismutase
TNF-α	Tumor necrosis factor-alpha
