# Supplementary material for: Quercetin Attenuates Pancreatic and Renal D-Galactose-Induced Aging-Related Oxidative Alterations in Rats
Source: Int J Mol Sci. 2020 Jun 18;21(12):4348. doi: 10.3390/ijms21124348 (PMC7352460; doi:10.3390/ijms21124348)
Supplement: Supplementary file 1 [file ijms-21-04348-s001.pdf]

Supplementary

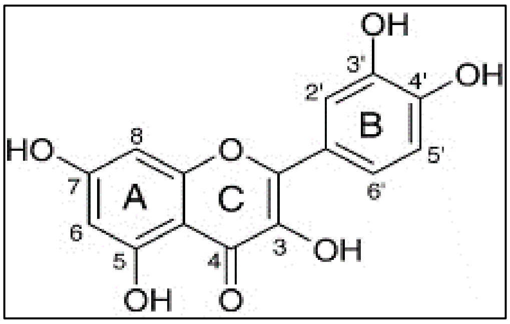

Figure S1. Chemical structure of quercetin.

Table S1. Ingredients of basal diet.

Ingredients	g/kg diet
Corn flour	529.5
Casein	200
Sucrose	100
Soybean oil	70
Cellulose	50
Mineral mix	35
Vitamin mix	10
L-cystine	3
Choline	2.5

Table S2. Antibodies, sources, working dilutions, and conditions for antigen retrieval (10 mM citrate buffer at pH 6.0).

Antibody	Source	Dilution	Heating Condition for 20 min
Rabbit polyclonal anti-Caspase-3	9662, Cell Signaling Technology, Danvers, MA, USA	1:300	105 °C
Rabbit polyclonal anti-Bcl2	SC-492, Santa Cruz Biotechnology, Santa Cruz, CA, USA	1:300	90 °C
Rabbit polyclonal anti-Ki67	GTX20833, GeneTex Co., Alton Pkwy Irvine, CA, USA	1:150	105 °C
Rabbit polyclonal anti-podocin	29040, IBL, Gunma, Japan	1:800	105 °C
Rabbit polyclonal anti-insulin	ab63820, Abcam, Cambridge, UK	1:500	105 °C
Rabbit polyclonal anti-glucagon	ab137817, Abcam	1:500	105 °C

Table S3. Primers' sequences for RT-PCR.

Genes	Genes Forward (5′–3′)	Reverse (5′–3′)	Accession No./References
Bax	GGCGAATTGGCGATGAACTG	ATGGTTCTGATCAGCTCGGG	NM_017059/[57]
Bcl2	GATTGTGGCCTTCTTTGAGT	ATAGTTCCACAAAGGCATCC	NM_016993/[58]
CASP-3	TTTGCGCCATGCTGAAACT	ACGAGTGAGGATGTGCATGAATT	NM_012922.2
GCLC	CCACTGTCCAAGGTTGACG	CTTGCTACACCCATCCACCA	NM_012815
GCLM	TCAAGCTCACAACTCA	CAAACCACCACATTCACGCC	NM_017305

IL-1β	CACCTCTCAAGCAGAGCACAG	GGGTTCCATGGTGAAGTCAAC	NM_031512.2/[59]
IL-6	CCACCAGGAACGAAAGTCAAC	TTGCGGAGAGAAACTTCATAGCT	NM_012589.2
IL-8	TGACCATGAGACACTGTGGC	GAAGAGCACGGGTCCTTTGA	[60]
NF-κB	TCTCAGCTGCGACCCCG	TGGGCTGCTCAATGATCTCC	AF079314
p21	GTGAGACACCAGAGTGCAAGA	ACAGCGATATCGAGACACTCA	[61]
p53	CACAGTCGGATATGAGCATC	GTCGTCCAGATACTCAGCAT	
SOD1	CATTCCATCATTGGCCGTACT	CCACCTTTGCCCAAGTCATC	BC082800.1
TNF-α	CAGCCGATTTGCCATTTCA	AGGGCTCTTGATGGCAGAGA	L19123.1
GAPDH (Housekeeping)	TCAAGAAGGTGGTGAAGCAG	AGGTGGAAGAATGGGAGTTG	NM_017008.4/[62]

Bax, Bcl-2-associated X protein; *Bcl-2*, B-cell lymphoma 2; *CASP-3*, caspase-3; *GCLC*, glutamate-cysteine ligase catalytic subunit; *GCLM*, glutamate-cysteine ligase regulatory subunit; *IL-1β*, interleukin-1beta; *IL-6*, interleukin-6; *IL-8*, interleukin-8; *NF-κB*, Nuclear factor-kappa B; *SOD1*, Cu, Zn-superoxide dismutase; *TNF-α*, tumor necrosis factor-alpha; and *GAPDH*, glyceraldehyde 3-phosphate dehydrogenase.
